# Self-Face Activates the Dopamine Reward Pathway without Awareness

**DOI:** 10.1093/cercor/bhab096

**Published:** 2021-04-16

**Authors:** Chisa Ota, Tamami Nakano

**Affiliations:** 1 Graduate School of Frontier Biosciences, Osaka University, Suita, Osaka, 565-0871, Japan; 2 Graduate School of Medicine, Osaka University, Suita, Osaka, 565-0871, Japan; 3 Center for Information and Neural Networks (CiNet), Suita, Osaka, 565-0871, Japan

**Keywords:** amygdala, self-face, subliminal, unconsciousness, VTA

## Abstract

The self-face advantage has been demonstrated not only at the supraliminal level, but also at the subliminal level. However, it remains unclear whether subliminal self-face processing involves the same neural networks as those for supraliminal self-face processing. Here, we show that the ventral tegmental area, a center of the dopamine reward pathway, exhibited greater activation to subliminal presentations of the self-face than those of the others’ faces, whereas subliminal presentations of the others’ faces induced activation in the amygdala, which generally responds to unfamiliar information. This self-other difference in brain response was consistently observed even when the facial configuration was modified without changing the shape of the facial parts. The present findings suggest that the dopamine reward pathway is involved in automatic self-advantage in face processing, and the subliminal self-other facial discrimination does not depend on information of the precise facial configuration.

## Introduction

The self-face has a special meaning to humans because of its importance for our identity and our sense of self. Correspondingly, the self-face has a cognitive advantage as it is processed more quickly and accurately than others’ faces (Tong and Nakayama 1999; Keyes and Brady 2010). This self-face prioritization effect also occurs compared with familiar faces (e.g., family and friends; Keyes et al. 2010; Bortolon and Raffard 2018). Therefore, this effect does not occur because your face is a highly learned face but because it is personally special information.

In addition to the behavioral evidence, neuroimaging studies using functional magnetic resonance imaging (fMRI) support the self-specialty in face processing. For example, the right-lateralized frontal and temporo-parietal regions showed greater activation to one’s own face than those of others (Sugiura et al. 2000; Uddin et al. 2005; Ota and Nakano 2021). Self-recognition has also been found to activate the cortical midline structures (Northoff 2016; Ota and Nakano 2021), which overlaps with the intrinsic default mode network (Raichle et al. 2001) and is involved in self-consciousness (Northoff et al. 2006).

Moreover, several subcortical regions have been reported to respond differently between the self-face and the other’s face. When one’s face looks more beautiful due to makeup, beauty retouching, or context, the dopamine reward pathway, such as the ventral tegmental area (VTA) and nucleus accumbens (NA) are activated, but this effect does not occur for others’ faces (Oikawa et al. 2012; Ueno et al. 2014; Ota and Nakano 2021). Instead, the amygdala, which generates a fear response, is activated to the unknown other’s face (Schwartz et al. 2003; Ota and Nakano 2021). Both self-face and self-related thoughts induced activation in the reward system including the VTA, NA, and the ventromedial prefrontal cortex (de Greck et al. 2008). Northoff and Hayes proposed that the fields of self and reward may benefit from increased interaction (Northoff and Hayes 2011).

**Figure 1:**
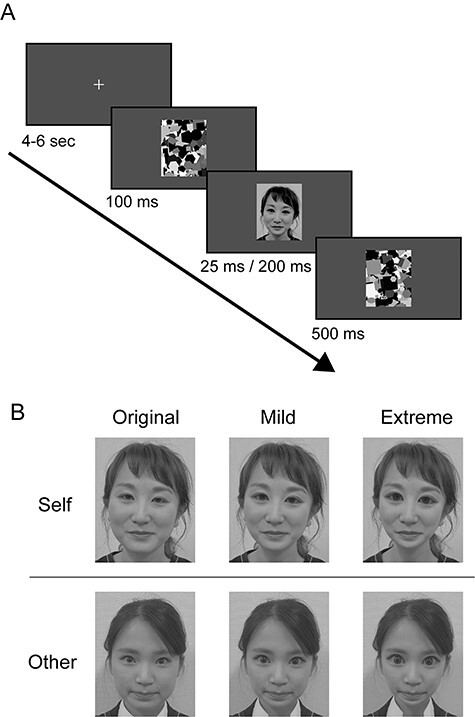
Experimental stimuli and procedures. (*A*) Protocol of the face evaluation task. After fixation, a face photo was presented for 25 or 200 ms (subliminal and catch trials, respectively) between the forward mask (100 ms) and backward mask (500 ms). (*B*) Examples of face images used in this study. Three filters (original, mild, and extreme) were applied to the self-face and other-face photos.

It is worth noting that this self-advantage is still observed without awareness. There has been an evidence that the subliminal presentation of the self-face automatically captures attention (Wojcik et al. 2019). An electroencephalogram (EEG) study has also shown that early neural markers in facial processing differ between one’s own face and other’s face when presented subliminally (Geng et al. 2012). This raises a question as to whether this subliminal self-face advantage involves the same brain areas that are activated by supraliminal presentation of the self-face, or whether different brain regions are involved. Despite accumulating evidence of self-face processing by neuroimaging studies, almost all of these investigations were pursued at the supraliminal level. The present study therefore used fMRI to examine the difference in brain activity between self-face and others’ faces when they were unconsciously presented by the forward–backward masking paradigm (Fig. 1*A*).

An additional question arises regarding what kind of information is utilized to discriminate the self-face from others’ faces without awareness. To address this issue, we also examined the effect of modification of facial configuration on the neural activity of subliminal self-other differences. In our recent study, we showed that a supraliminal presentation of mildly modified self-face by using a beauty filter, which made the eyes larger and the chin narrower without changing the shape of facial parts, activated the NA in addition to the self-relevant brain regions (Ota and Nakano 2021). By contrast, an extreme facial modification, which resulted in making the self-face “creepy,” no longer activated the self-relevant brain regions or the NA, but instead induced activation in the amygdala. These findings suggest that the extremely modified self-face is no longer recognized as a self-face. However, no study has investigated whether a subliminal face modification influences the brain activity of self-other discrimination. In the present study, we therefore subliminally presented faces whose configuration was mildly or extremely modified in addition to the original faces (Fig. 1*B*), and examined effects of face modification on the brain activity related to subliminal self-other facial discrimination.

## Materials and Methods

### Participants

Twenty-two women participated in this study (mean age: 22.6 years and range: 20–25 years). They had no abnormal neurological history and had normal vision either uncorrected or corrected by glasses. We focused on women because the photo retouching software that we used in this study is developed for women. The review board of Osaka University approved the experimental protocol (FBS30-4), and our procedures followed the guidelines outlined by the Declaration of Helsinki. All participants provided written informed consent prior to the experiment.

### Stimuli and Experimental Procedure

Before the fMRI experiment, we took a picture of each participant’s face as they stood in front of a white background with a black cape across their shoulders. For each participant, we took pictures of 10 different facial expressions without emotion (frontal face, face uttering “a,” “i,” “u,” “e,” “o” and faces tilted to the right, left, up, and down). These photos were used as self-face stimuli. In addition, we took pictures of 10 different facial expressions for 13 women (mean age: 22.2 years and 20–25 years old), who were not acquainted with the fMRI participants. Ten women’s photos were used for other-face stimuli, and 3 women’s photos were used for stimuli in the catch trial. Next, a beauty filter application (free software SNOW, Snow Corp.) that enlarges the eyes and irises and narrows the chin was applied to these photos in 2 stages: mild and extreme (Fig. 1*B*). A mild face was created by enlarging the eyes of the original face by 1.1 times, enlarging the iris by 1.1 times, and reducing the width of the lower chin by 0.9 times. The extreme face was created by enlarging the eyes of the original face by 1.21 times, enlarging the iris by 1.21 times, and reducing the width of the lower chin by 0.81 times. Since the size of the iris has been further enlarged, the modified face has larger pupils and more widely eyes than the original face. For each participant, we prepared a series of self-face and other-face images with either no filter, a mild filter, or an extreme filter applied. All photo images were 400 × 500 pixels in size and converted to gray scale with mean intensity, with mean intensity adjusted to 125 and a standard deviation (SD) of intensity set to 40. The mask image (gray scale, 400 × 500 pixels) was created by superimposing various sizes and luminance of polygons (triangles to pentagons) and ovals using Psychophysics Toolbox Version 3 (PTB-3) and Matlab (Mathoworks, Inc.).

During the experiment, the participants laid in a magnetic resonance imaging (MRI) scanner while wearing earplugs and immobilizing their heads using sponge cushions, and viewed visual stimuli on a screen (1920 × 1080 pixels, refresh rate 120 Hz, and viewing angle = 27.1°) via a mirror placed in front of their eyes. In each trial, following the presentation of the fixation cross for 4–6 s with a gray background, a forward mask image was presented for 100 ms, and the face photo was subsequently presented for 25 ms (Fig. 1*A*). Afterwards, a backward mask image was immediately presented for 500 ms. A combination of the brief presentation of the face stimuli and visual masking of salient images prevents participants from being aware of the presentation of the facial images (subliminal stimuli). The presentations of the stimuli were controlled by the Presentation (Neurobehavioral Systems Inc.). In order to confirm whether the face image was subliminal, the participants were asked to press a button as soon as they perceived a face in each trial. An MRI-compatible 2-button response device was placed under their right index finger and middle finger (they can use either button). In order to check their engagement in the task, catch trials were randomly interposed (14% of trials), in which the face image was presented for 200 ms instead of 25 ms. Twelve trials were presented for each face type and catch trial, for a total of 84 trials in 1 session. The order of the face stimuli was randomized across the participants. All participants completed 3 sessions, with a short break between sessions, for a total of 252 trials. Different facial images were used in each session of the other-face.

### Data Acquisition

Functional images were acquired using multiband T2*-weighted gradient echo-planar imaging (EPI) sequences, which were obtained using a 3-Tesla MRI scanner (MAGNETOM Vida, Siemens), with a 64-channel array coil. We collected 490 scans per session (slice number = 45, slice thickness = 3 mm, repetition time [TR] = 1000 ms, echo time [TE] = 30 ms, flip angle = 60°, field of view [FOV] = 192 × 192 mm, voxel size [*x*, *y*, and *z*] = 3 × 3 × 3 mm, and multiband factor = 3). For an anatomical reference image, a T1-weighted structural image was acquired for each subject (magnetization prepared rapid gradient echo sequence, slice thickness = 1 mm, TR = 1900 ms, TE = 3.37 ms, flip angle = 9°, FOV = 256 × 256 mm, and voxel size [*x*, *y*, and *z*] = 1 × 1 × 1 mm). To correct the geometric distortion in EPI, we also acquired field maps for each participant (Siemens standard double echo gradient echo field map sequence, slice thickness = 3 mm, TR = 753 ms, TE = 5.16 ms, flip angle = 90°, FOV = 192 × 192 mm, and voxel size [*x*, *y*, and *z*] = 3 × 3 × 3 mm).

### Imaging Data Analysis

Acquired MRI data were processed using SPM12 and MATLAB2019a. We discarded the first 3 EPI images in each session. To correct image distortion due to field inhomogeneity, field map correction was applied using the SPM fieldmap toolbox. We confirmed that the head movements were < 3 mm in all participants, and the EPI images were realigned and unwarped. Each participant’s structural image was coregistered to the mean of the motion-corrected functional images. Subsequently, the EPI images were normalized to the standard brain template (Montréal Neurological Institute template), and smoothed using a Gaussian kernel filter with an 8-mm full-width-at-half-maximum. After preprocessing, we conducted a voxel-by-voxel regression analysis of expected hemodynamic changes for the 6 conditions (self/other × 3 filter level) using the general linear model (GLM) on the single-subject level. To capture small offsets in the time to peak of the response, the canonical hemodynamic response function and its first derivative approaches were applied to a GLM. We also regressed out global signal due to motion artifacts using realignment parameters. Finally, the design matrix consisted of 6 conditions of face stimuli and button press, and 6 realignment parameters per session. Trials in which the participants reported perceiving the face image were excluded from the analysis. On the group level, the 2-way ANOVA was performed with factors for face type (self/other) and filter level (original, mild, and extreme). For whole brain analyses, we used a family wise error rate (FWE) cluster-corrected threshold of *P* < 0.05, using a cluster-defining threshold of *P* < 0.001. Based on prior work, we hypothesized that the cortical and subcortical regions that show differences in activity between the self- and other-faces at the supraliminal level are also involved in self-other discrimination at the subliminal level. Therefore, we used a small-volume corrected FWE cluster-level threshold of *P* < 0.05 in spheres with a 10-mm radius around previous coordinates in the amygdala (right [27, −4, −19] and left [−27, −10, −19]; Ota and Nakano 2021), VTA [3, −16, −12] (Trutti et al. 2021), NA (right [12, 8, −7] and left [−15, 5, −10]), inferior frontal gyrus (IFG; [48, 29, 5]), superior marginal gyrus (SMG, right [63, −22, 23], left [−63, −37, 32]), anterior cingulate cortex (ACC; [0, 26, 23]), and posterior cingulate cortex (PCC; [12, −31, 41]; Ota and Nakano 2021).

## Results

We first analyzed the proportion of trials in which participants were aware of the presentation of face stimuli. The face perception rate was very low when the face stimuli were presented for 25 ms (subliminal condition, self-face; original 1.6 ± 0.70%, mildly filtered 1.5 ± 0.75%, extremely filtered 2.7 ± 1.2%, other-face; original 1.1 ± 0.39%, mildly filtered 1.9 ± 0.85%, extremely filtered 2.5 ± 0.77%, mean ± SD), but near 100% when they were presented for 200 ms (catch trials, 97 ± 1.7%). A 2-way analysis of variance (ANOVA) showed that neither face type (self/other) nor the filter level (original, mild, and extreme) affected the perception rate in the subliminal condition (face type *F*_1,21_ = 0.047, *P* = 0.83; filter level *F*_2,42_ = 2.16, *P* = 0.13). Subsequent imaging analyses used “subliminal” trials in which participants were not aware of the presentation of face images in the subliminal conditions.

**Figure 2:**
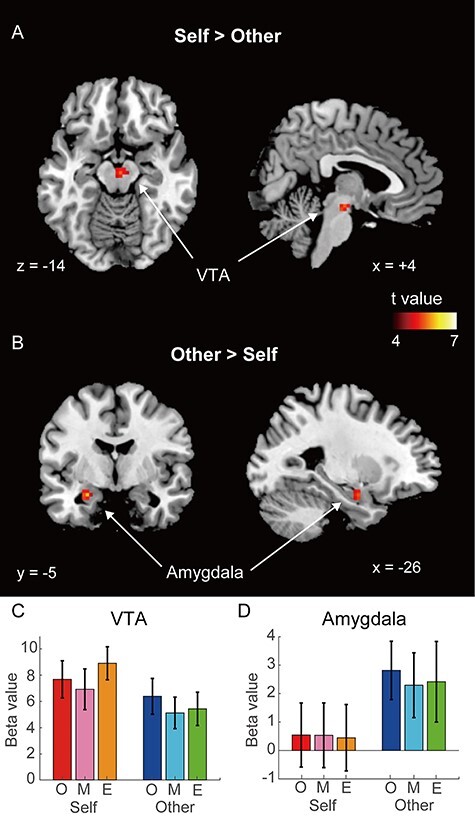
Brain regions exhibiting a self-other difference. (*A*–*B*) Brain regions showing greater activation to the self-face than other-face (*A*), and to the other-face than self-face (*B*). (FWEc *P* < 0.05, with small volume correction, voxel-level *P* < 0.001). The color bars represent voxel-level *t*-values. The mean beta value of the VTA (*C*) and amygdala (*D*) for each condition. In the horizontal axis, “O,” “M,” and “E” represent the original, mild, and extreme filters, respectively. The error bars represent the standard error.

Next, we analyzed brain activity evoked by the subliminal facial presentations using the 2-way ANOVA with factors of face type and filter level. No significant main effect or interaction was observed in the whole-brain analysis (FWEc, *P* < 0.05; single voxel, *P* < 0.001). However, the ROI-based analysis identified a significant main effect for face type but no significant main effect for filter level and no significant interaction. Post-hoc test revealed that the VTA exhibited significantly greater activation to the self-face than the other-face (Fig. 2*A*, MNI coordinates *x* = 3, *y* = −16, *z* = −13, *k* = 15, *t* value = 4.6, FWEc *P* < 0.05, with small volume correction). In contrast, the left amygdala exhibited greater activation to the other-face than the self-face (Fig. 2*B*, MNI coordinates *x* = −24, *y* = −4, *z* = −19, *k* = 11, *t* value = 5.3, FWEc *P* < 0.05, with small volume correction). Even when applying the ROI-based analysis, the cortical regions did not show any significant self-related activity. We further analyzed the mean beta values of these brain regions. The mean beta value in the VTA was higher for all filter levels of self-face than those of other-face, while that in the amygdala was higher for all filter levels of other-face than for self-face (Fig. 2*C* and *D*). The distributions of individual beta values for each face type in the VTA and amygdala are shown in Supplementary Fig. S1. Eighteen of the 22 participants consistently showed higher beta values in the VTA and lower beta values in the amygdala for self-face than for other-face.

## Discussion

The present study examined the brain regions involved in unconscious self-other distinction using a brief presentation of face images with forward–backward masks. The face detection rate did not differ between the self-face and others’ faces at the behavioral level, but the brain responded completely differently to the subliminal presentation of the self-face from those of others’ faces. The VTA responded more to the self-face than to others’ faces, and the amygdala responded more to others’ faces than to the self-face. This self-other difference in brain response was consistently observed even in the extremely modified face. These results address important topics related to the neural mechanism underlying the self-face advantage, the different neural processing of self-face between the supraliminal and subliminal levels, and the mechanisms of unconscious self-other discrimination.

### Neural Mechanisms Underlying the Self-Face Advantage

The self-face advantage, automatically grabbing attention and being processed quickly and accurately, was consistently observed at both the supraliminal and subliminal levels (Tong and Nakayama 1999; Keyes and Brady 2010; Bortolon and Raffard 2018; Wojcik et al. 2019). Studies examining neural activity also showed that presentation of the self-face enhances face-specific event-related potential (ERP) components (Geng et al. 2012) and induces additional activation in various cortical regions (Sugiura et al. 2000; Uddin et al. 2005). However, the mechanism that prioritizes or biases the neural processing of the self-face remains unclear. The present study found that the VTA increased neural activity in response to the self-face even when the participants were not aware of the presentation of their own face. The VTA located in the midbrain is the center of the reward pathway and sends dopaminergic neural projections to the NA and prefrontal cortex (Haber and Knutson 2010). The dopamine signals arising from the VTA carry value-based information to the neurons in layer 5 of the cortex and enhance and prioritize neural processing accordingly (Thiele and Bellgrove 2018). Berke proposed that dopamine provides a dynamic estimate of whether it is worth expending limited internal resources such as attention (Berke 2018). From this point of view, the automatic attention capture to the subliminal presentations of self-face (Wojcik et al. 2019) can be explained as a result of dopaminergic modulation. Therefore, the present study provides a new perspective on the neural mechanisms underlying the self-face advantage: since the self-face has a positive reward value for oneself, the VTA is automatically and instantaneously activated by the presentation of the self-face even without awareness. This dopaminergic modulation from the VTA prioritizes and enhances the cortical processing of self-face information. As a result, a self-face advantage would be observed at the behavioral level.

One might argue that different numbers of repeated presentations between self-face and other faces induce a difference in brain activity between them. However, a previous study clearly demonstrated that repeated subliminal presentations of unfamiliar faces do not induce a prioritization effect that is observed in the self-face (Bola et al. 2021). Therefore, we speculate that VTA activation in response to the self-face is not caused by the effect of repetitive presentations of the face. Thus, the question arises that face familiarity may have induced the activation of the reward pathway. However, even compared with familiar faces such as those of family and friends, the priority effect of self-face is consistently observed (Keyes and Brady 2010; Bortolon and Raffard 2018). In addition, there are no reports that familiar faces induce activation in the reward pathway when it is supraliminally presented, whereas there are several evidences that the self-face does it (Oikawa et al. 2012; Ota and Nakano 2021). Moreover, not only self-face but also self-related information induced neural activity in the reward system (de Greck et al. 2008). Considering these evidences, we assume that the VTA activation in response to the subliminal presentation of the self-face cannot be simply explained by the factor of face familiarity. Further studies are expected to examine this possibility by comparing brain activity while face familiarity is controlled.

Another issue of the present study is whether there is a gender difference in activity of the reward system to the self-face. The present study recruited only female participants to apply the face filter for female faces. The previous studies that reported the VTA activation to the supraliminal self-face also recruited only female participants (Oikawa et al. 2012; Ota and Nakano 2021). Therefore, it is unclear whether the reward system is activated by the self-face even in male. Since the self-face advantage is consistently observed in both men and women, we assume that the same neural system might work not only for female but also male. Future study is necessary for investigating the neural activity in response to self-face inmale.

### Differences in Neural Processing of Self-Face Depending on the Levels of Awareness

By looking at pictures or mirror images of the self-face, we objectively recognize our external appearance and internal mental states and update representation of the self-image in the brain accordingly. This process is called self-awareness. When the face images are presented at the supraliminal level, the self-face induces activation not only in face-related brain areas (e.g., the fusiform gyrus and the occipital face area), but also in the right inferior frontal gyrus (IFG), the temporo-parietal junctions, and the medial cortical structures (Sugiura et al. 2000; Uddin et al. 2005; Northoff et al. 2006; Ota and Nakano 2021). The right IFG and temporo-parietal junctions exhibited self-specialty processing limited to facial information (Uddin et al. 2005), but the medial cortical structures exhibited activation in response to various self-related information (Northoff et al. 2006). Therefore, the medial cortical structure is suggested to be involved in the formation of general self-awareness. It is also supported by the evidence that the degree of consciousness in patients with disordered consciousness was correlated with neural activity in these regions induced by self-related stimuli (Qin et al. 2010; Huang et al. 2014). Both self and reward information are represented in the anterior part of the ventromedial prefrontal cortex when self-information is presented supraliminally (Yankouskaya et al. 2017).

In contrast, the present study revealed that these self-related cortical areas did not show any activation to the self-face when presented subliminally. Consistently, a previous EEG study reported a different self-related modulation of the face ERP component between subliminal and supraliminal presentations (Geng et al. 2012). The early face component exhibited a self-other difference at the subliminal level, while the late face component exhibited it only at the supraliminal level. Panksepp and Northoff (2009) proposed that the sense of self consists of several hierarchical structures: proto-self, core-self, and higher-order of self-representation. Based on this concept, the present findings suggest that the lower order of self-processing automatically occurs without awareness, while the higher-order of self-processing involving the cortical medial structures and the frontal and temporo-parietal regions require conscious awareness of the self-face. It is worth noting that the explicit feeling of self-relatedness is influenced by the neural activity in the subcortical regions including the ventral striatum and amygdala (Schneider et al. 2008) and self-related processing occurs via integration between subcortical and cortical medial structures (Panksepp and Northoff 2009). Therefore, our sense of self might be completed by integration of the information between the hierarchical structures of proto-self, core-self, and higher-order self-processing.

### Mechanisms of Self-Other Discrimination without Awareness

As a prerequisite for producing a self-face priority effect, it is necessary to distinguish the self-face from another’s face. In the brain, face-specific neural networks, including the fusiform face area and the occipital face area, discriminate and identify the face by analyzing the facial configuration and the shapes of facial parts (Kanwisher et al. 1997; Yovel and Kanwisher 2004). It then raises a question as to how the brain discriminates the self-face from the other’s face without awareness. In our previous study that supraliminally presented the same face stimuli, we showed that the extremely modified faces did not activate the self-face specific cortical areas (Ota and Nakano 2021). The results suggested that an extremely modified self-face is no longer recognized as self-face. In contrast, when the extremely modified face was presented subliminally in the present study, the self-other difference in brain response was consistently observed. Assuming that the beauty filter application largely transformed the face configuration by changing the relative size of facial parts, the face configuration is critical information for supraliminal self-other discrimination. On the other hand, given that even the extremely modified faces were correctly discriminated in the subliminal condition, it is likely that the subliminal self-other facial discrimination does not depend on the facial configuration. This is supported by a previous EEG study comparing the self-face specialty in ERP between the subliminal and supraliminal levels (Geng et al. 2012). This study reported that the N170 component, which reflects the encoding of facial configuration for face identification, is larger for the self-face compared with the other’s face when presented supraliminally, but no difference between the self- and other faces was observed when presented subliminally. Combined with another finding of the present study that the amygdala, which reacts to unfamiliar face stimuli (Schwartz et al. 2003), exhibits greater activation to the subliminal presentation of the unknown other’s face, we assume that unconscious self-other discrimination is based on the information that the facial parts are familiar or novel for oneself.

The next question arises whether this self-other discrimination is processed at the cortical or subcortical level. It is well known that the subcortical visual pathway from the superior colliculus to the pulvinar is responsible for fast and coarse facial processing (Tamietto and de Gelder 2010). This pathway analyzes holistic facial patterns based on low-resolution information of the face and enables the quick reaction of human faces (Nakano et al. 2013). However, the present findings revealed that unconscious self-other discrimination occurred even when the holistic facial pattern was transformed. Consistent with this finding, previous studies have reported that the self-face advantage even occurs in the inverted face (Bortolon and Raffard 2018). Given that the subliminal face stimuli induce the activation of face-related cortical regions (Morris et al. 2007) and the subliminal self-related stimuli (name, birthday, and nationality) also activate the interior temporal and fusiform gyrus (Tacikowski et al. 2017), it is possible that the cortical pathway may be involved in self-other discrimination. These assumptions, however, need to be further explored in future studies. For example, it is necessary to investigate whether it is possible to discriminate between self and others only with subliminal presentation of facial parts, and which spatial frequency of the facial information are used to unconsciously discriminate between self and others by applying a spatial filter.

## Limitations

The present study simply analyzed the brain response to visual stimuli using the general linear model, but the various methods analyzing functional imaging data are proposed. For example, functional connectivity analyses can examine task-related regional interactions. Several studies also reported that spontaneous brain activity influences task-related brain activity (Huang et al. 2017; Scalabrini et al. 2019). This suggests that temporo-spatial dynamics produced by spontaneous brain activity are the basis of both neuronal and phenomenal states (Northoff and Lamme 2020). However, since the duration of facial stimuli was very short in this study (25 ms), we set very short inter-trial intervals to increase the number of trials for robust measurement of the brain response. This makes it difficult to stably analyze the functional connectivity between regions by task and the correlation between task and rest states. Future studies are expected to investigate subliminal self-face processing using these analyses.

In summary, our studies advance the understanding of the neural mechanisms of subliminal self-face processing in several aspects. First, the dopamine reward pathway is automatically involved in unconscious self-face processing. This suggests that dopaminergic neuromodulation produces a self-face advantage in both behavioral and neural processing. Second, the cortical areas related to the higher-order of self-consciousness are not involved in subliminal face processing. Finally, the modulation of the facial configuration does not affect the self-other discrimination at the subliminal level, and the amygdala consistently exhibits activation to the unknown others’ faces. These results suggest that the mechanisms enabling subliminal self-other discrimination depend on neural encoding of familiarity and novelty of the facial parts.

## Funding

This work was supported by Grant-in-Aid (18H04084, 18H05522 awarded to T.N.) by the Ministry of Education, Culture, Sports, Science, and Technology, Japan, as well as a PRESTO grant, “The Future of Humans and Interactions” (#30227, awarded to T.N.) by the Japan Science and Technology Agency, Japan.

## Notes

We would like to thank Dr Shigeru Kitazawa for valuable comments on our study. *Conflict of Interest:* None declared.

## Supplementary Material

Supplementary_Figure_1_bhab096
