# Supplementary figures and images for: Self-Face Activates the Dopamine Reward Pathway without Awareness
Source: Cereb Cortex. 2021 Apr 16;31(10):4420–6. doi: 10.1093/cercor/bhab096 (PMC8408479; doi:10.1093/cercor/bhab096)

Supplementary Figure 1.

Distributions of mean beta value for each face type in VTA and amygdala.

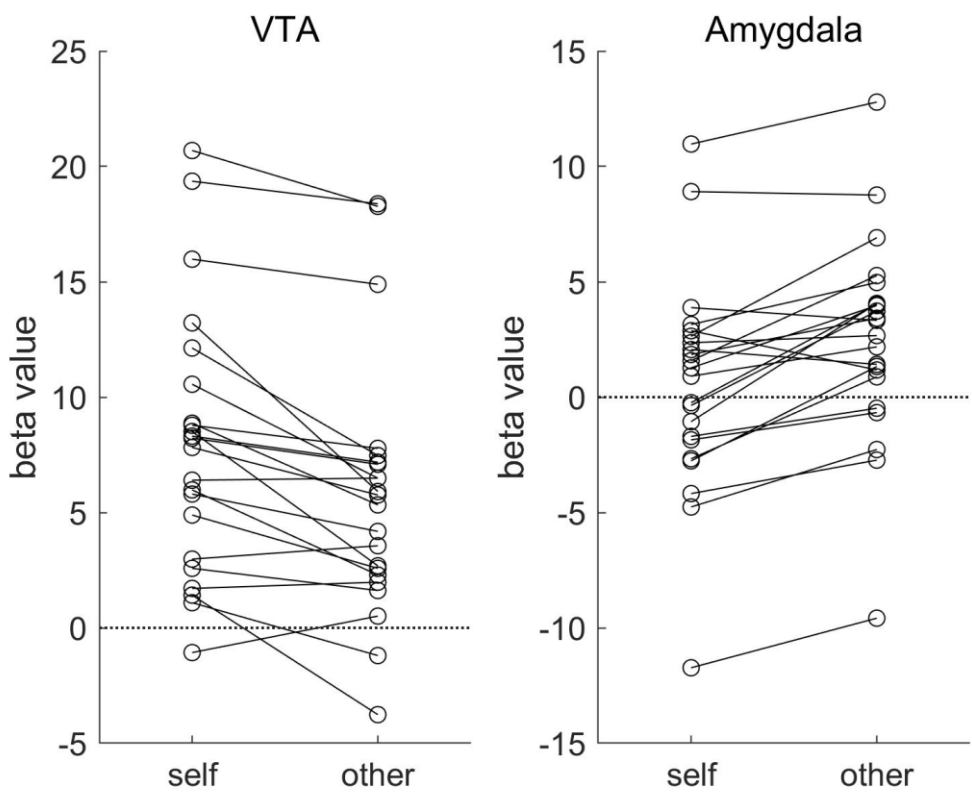

Supplement: Supplementary_Figure_1_bhab096 [file supplementary_figure_1_bhab096.pdf]
